# Improved Energy Storage Performance of All-Organic Composite Dielectric via Constructing Sandwich Structure

**DOI:** 10.3390/polym12091972

**Published:** 2020-08-31

**Authors:** Mengjia Feng, Tiandong Zhang, Chunhui Song, Changhai Zhang, Yue Zhang, Yu Feng, Qingguo Chi, Qingguo Chen, Qingquan Lei

**Affiliations:** 1 Key Laboratory of Engineering Dielectrics and Its Application, Ministry of Education, Harbin University of Science and Technology, Harbin 150080, China; mjfeng_ma17@hrbust.edu.cn (M.F.); songchunhui@hrbust.edu.cn (C.S.); chzhang@hrbust.edu.cn (C.Z.); yzhang_ana@sina.com (Y.Z.); qgchi@hotmail.com (Q.C.); qgchen@263.net (Q.C.); lei_qingquan@sina.com (Q.L.); 2 School of Electrical and Electronic Engineering, Harbin University of Science and Technology, Harbin 150080, China

**Keywords:** sandwich structure, energy storage efficiency, PMMA, P(VDF-TrFE-CFE)

## Abstract

Improving the energy storage density of dielectrics without sacrificing charge-discharge energy storage efficiency and reliability is crucial to the performance improvement of modern electrical and electronic systems, but traditional methods of doping high-dielectric ceramics cannot achieve high energy storage densities without sacrificing reliability and storage efficiency. Here, an all-organic energy storage dielectric composed of ferroelectric and linear polymer with a sandwich structure is proposed and successfully prepared by the electrostatic spinning method. Additionally, the effect of the ferroelectric/linear volume ratio on the dielectric properties, breakdown, and energy storage is systematically studied. The results show that the structure has good energy storage characteristics with a high energy storage density (9.7 J/cm^3^) and a high energy storage efficiency (78%). In addition, the energy storage density of the composite dielectric under high energy storage efficiency (90%) is effectively improved (25%). This result provides theoretical analysis and experience for the preparation of multilayer energy storage dielectrics which will promote the development and application of energy storage dielectrics.

## 1. Introduction

Dielectric capacitors are widely used in pulsed power electronics and new energy vehicles due to their high power density and ultrashort discharge time [1,2,3,4]. Increasing the energy storage density of the dielectric is beneficial to the miniaturization of capacitors and the optimization of related systems [5]. The energy storage density of the dielectric is calculated by the following formula: $U_{e}=\int EdD$, where *U*_e_ is the energy storage density, *D* is the electrical displacement, and *E* is the applied electric field strength [5]. *D* is proportional to the relative dielectric constant $\varepsilon_{r}$. Polymers have received a lot of attention due to their high breakdown and good processability. The current commercially available capacitor dielectric is biaxially oriented polypropylene (BOPP), which has an energy storage density of 2 J/cm^3^, with ultra-high energy storage efficiency and reliability. However, the required properties for capacitor dielectrics, such as high energy storage density, high breakdown strength, and low dielectric losses, are often difficult to obtain in a polymer material at the same time. Therefore, for a long time, the optimization method of dielectric energy storage performance has been doping inorganic ceramic with high $\varepsilon_{r}$ into polymers to prepare the inorganic-organic composite dielectric, and some achievements have been made [2,6,7,8,9,10,11]. However, due to the huge gap in dielectric properties and electrical conductivity between inorganic ceramics and polymers, electric field distortions are easily formed at the inorganic-organic interface, which may lead to deterioration of breakdown performance [12,13]. Additionally, the dielectric loss of ceramic fillers is relatively high, which reduces the energy storage efficiency of the dielectric. Therefore, it is necessary to develop new dielectric design methods to increase the energy storage density of the dielectric without sacrificing energy storage efficiency and reliability [14,15,16].

To solve the above problems, the preparation of polymer-polymer composite dielectric is considered [17,18,19,20,21]. It is difficult to control the interface and crystal regions in blend films, resulting in greater difficulty in improving performance [22,23]. Compared with blend films, in multilayer dielectrics, the performance can be adjusted by controlling the thickness ratio and arrangement of different layers [22,24,25,26,27,28]. The interfaces perpendicular to the direction of the electric field in the multilayer dielectric are beneficial for increasing the energy barrier of carrier movement [25,29,30,31]. The interface can increase the tortuosity of the breakdown path to increase the breakdown strength [32]. There are many studies on improving energy storage performance by establishing sandwich structure, some of which have achieved remarkable progress [10,33,34,35,36,37,38,39]. Theoretically, by adjusting the thickness and position of interfaces the energy storage characteristics of the composite dielectric can be adjusted. However, the effect of the three-layer thickness ratio on energy-storage performance is rarely studied, which is crucial to establish and understand the theoretical models for hierarchical structure dielectric.

To fill the theoretical gap mentioned above, in this study the linear polymer polymethyl methacrylate (PMMA) and the ferroelectric polymer P(VDF-TrFE-CFE) (PVTC) were employed to prepare the sandwich-structure composite dielectrics. The dielectric, energy storage, and breakdown characteristics of dielectrics with different thickness ratios were investigated. P(VDF-TrFE-CFE) is a semicrystalline terpolymer comprising of vinylidene fluoride (VDF), trifluoroethylene (TrFE), and 1,1-chlorofluoroethylene (CFE). The addition of defects in the form of TrFE and CFE molecules in the polymer chain serves to interrupt the ferroelectric domain, thereby reducing its size [40]. Polymethyl methacrylate (PMMA), or plexiglass, is a transparent polar non-ferroelectric polymer [41,42]. The PVTC layer (with high dielectric constant) is the polarization layer which provides the polarization, and the PMMA layer (with high breakdown strength and low leakage current) is the breakdown layer which bears a bigger electric field. The structure is expected to combine the advantages of PVTC and PMMA to achieve excellent energy storage performance. In addition, PMMA is compatible with PVTC and they have different melting points, which is beneficial for successful preparation of sandwich structure. Note that the other parameters remain the same while changing the thickness ratio.

A series of sandwich-structured dielectrics with PVTC and PMMA volume ratios of 1:9, 3:7, 5:5, 7:3, and 9:1, which are denoted as 1-9, 3-7, 5-5, 7-3, and 9-1 in the following text, were prepared and their dielectric, breakdown, and energy-storage characteristics were systematically studied. The results showed that the composite dielectric has a high energy storage density of 9.7 J/cm^3^ when the volume ratio of PVTC is 10 vol %. In addition, the 1-9 sandwich-structure PVTC/PMMA composite dielectric is able to maintain an energy storage density of 2.5 J/cm^3^ when the energy storage efficiency is 90%, which is 25% higher than BOPP. The study of sandwich-structured composite dielectric with outstanding energy storage properties could be a potential direction in the field of energy storage.

## 2. Experimental Section

### 2.1. Material and Methods

PVTC powder was provided by Dupont (Wilmington, DE, USA). The average molecular weight and the molar ratio are 200,000 and 60/20/20, respectively. PMMA powder was provided by Arkema (Paris, France) and the average molecular weight is 12,000. The organic solvent *N*,*N*-dimethyl formamide (DMF) (99.0%), was provided by Sinopharm Chemical Reagent Co., Ltd (Shanghai, China).

**Scheme 1:**
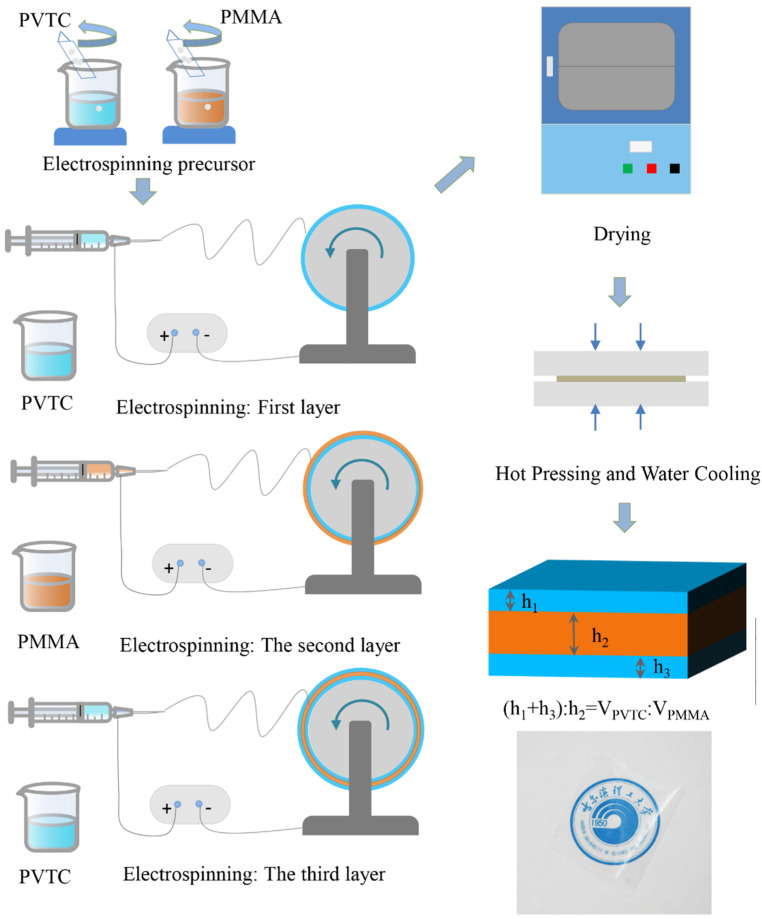
Preparation scheme for sandwich-structure composite dielectrics by electrospinning and hot-pressing method.

As shown in Scheme 1, a series of sandwich-structure dielectrics were prepared by electrospinning technology. PMMA powder was added into organic solvent (DMF) in a ratio of 2 g: 10 mL, and the powder was fully stirred until it was completely dissolved to form a clear solution. The PVTC powder was added into organic solvent (DMF) in a ratio of 1 g: 10 mL, and the powder was fully stirred until it was completely dissolved and prepared into a clear solution. The solution was put into a 10 mL syringe. The needle type of the syringe was 23G, and an appropriate amount of PVTC solution and PMMA solution was extracted with the syringe in accordance with the requirements and spun on electrostatic spinning equipment. The volume of the precursor was calculated by the volume ratio of PVTC and PMMA and is shown in Table 1. The distance between needle and collector was 16 cm. The spinning sequence was PVTC, PMMA, and PVTC. Propulsion speed of the syringe was set at 2 mm/min, the receiver speed was 100 r/min, the positive voltage V_+_ at the syringe needle was 10 kV, and the negative voltage V_−_ at the receiver was 10 kV. After the end of spinning, the precast fibers were in a low humidity state, which was put into a high temperature air-blast drying oven for 6 h. Then, the dry dielectric was hot-pressed with a plate vulcanizing machine at a temperature of 175 °C and a pressure of 15 MPa, and the hot-pressed time was 5 min. It is worth noting that in order to prevent the diffusion at the interface, the parameters of hot pressing, namely hot-pressing temperature and hot-pressing time, need to be controlled and a temperature (175 °C) at which PMMA melts but PVTC does not melt was used. The sandwich-structure composite dielectric was obtained after water cooling to room temperature. The total thickness of the pristine PVTC and sandwich-structure composite dielectric were both 24–27 μm and the thickness of PMMA was 22–24 μm.

### 2.2. Microstructure and Properties Characterization

The following devices were used for the microstructure characterization: JASCO 6100 (Tokyo, Japan) for Fourier Transform Infrared (FTIR) and Hitachi SU8020 Uhr (Tokyo, Japan) for field emission scanning electron microscope (FE-SEM). The following devices were used for properties characterization: GmbH Novocontrol Alpha-A (Montabaur, Germany) for recording dielectric properties and Radiant Premier II Ferroelectric Test System with a High Voltage Power Supply for recording unipolar ferroelectric hysteresis loops and breakdown strengths. Triangulation waves of 100 Hz are used in ferroelectric testing.

## 3. Results and Discussion

**Figure 1:**
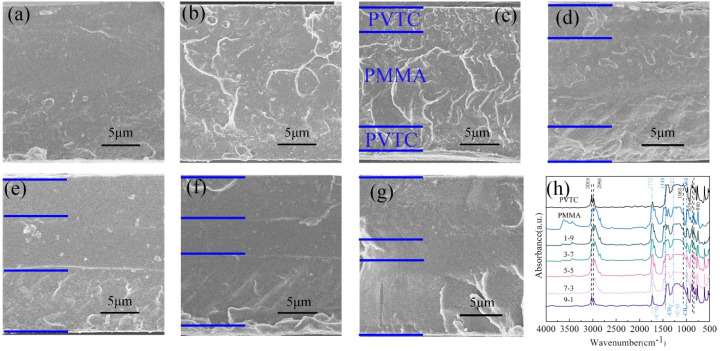
SEM images for (**a**) PVTC, (**b**) PMMA, (**c**) 1-9, (**d**) 3-7, (**e**) 5-5, (**f**) 7-3, and (**g**) 9-1 sandwich-structure composite dielectric. (**h**) FTIR spectra for pristine PVTC, PMMA, and sandwich-structure composite dielectric with different ferroelectric/linear volume ratio.

Figure 1a–e shows cross-sectional electron micrographs of PVTC, PMMA, and sandwich-structured PVTC/PMMA composite dielectric. The interior of the dielectric is uniform and dense, with no visible defects. It is worth noting that in order to prevent the diffusion at the interface, the parameters of hot pressing, namely hot-pressing temperature and hot-pressing time, need to be controlled, and a temperature (175 °C) at which PMMA melts but PVTC does not melt was used. Nevertheless, some interfaces could not be clearly observed, which may have been caused by the excellent compatibility between PVTC and PMMA. FTIR was employed to investigate the microstructure structure of the pristine PVTC, PMMA, and sandwich-structure composite dielectric. Figure 1 shows the infrared characteristic absorption peaks of -O-CH_3_ bending vibration. Stretching vibration in PMMA was found near 989 and 1449 cm^−1^, and infrared characteristic absorption peaks of stretching vibration O=C in PMMA near 1272 and 1732 cm^−1^ were also found [37,43]. The infrared characteristic absorption peaks of C-F_2_ in PVTC were found near 840 and 879 cm^−1^. The oscillatory infrared characteristic absorption peaks of C–H_2_ in PVTC were found near 1065 cm^−1^ [44,45]. According to the FTIR spectra, it can be seen that no new substances were formed during the preparation process. In short, a series of sandwich-structure PVTC/PMMA composite dielectric with different volume ratios were successfully prepared.

**Figure 2:**
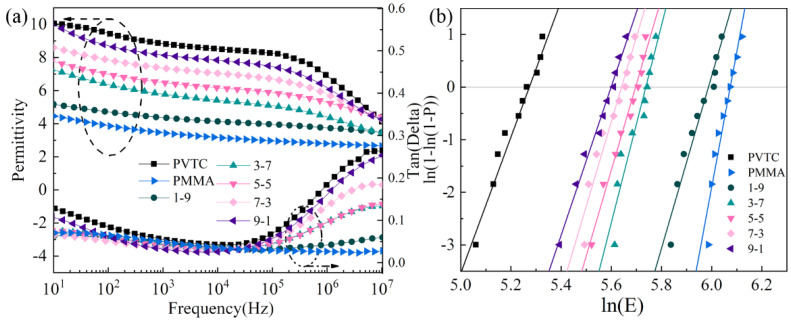
(**a**) Permittivity and dielectric loss for pristine PVTC, PMMA, and sandwich-structure dielectrics and (**b**) Weibull distribution of breakdown strength.

Permittivity is one of the most important parameters for energy storage dielectrics [2]. The dielectric properties are shown in Figure 2a. As can be seen from the figure, the permittivity of all dielectrics decreases as the frequency increases in the range of 10^1^ Hz to 10^7^ Hz. The first thing that can be observed is that the permittivity of sandwich-structure composite dielectric lies between pristine PVTC and PMMA. The permittivity of the sandwich-structure composite dielectric increases with the increase of the volume fraction of PVTC, which is consistent with the capacitor series model [7,46]. Additionally, the dispersion in 9-1 composite dielectric is most obvious. The rising trend of permittivity of 9-1 composite dielectric in the low frequency region reflects the existence of interface polarization, which can increase the polarization to some extent [47,48,49,50].

The breakdown strength is another important factor affecting the energy storage performance. In this work, the breakdown strength of dielectrics was tested, and the results are shown in Figure 2b. The two-parameter Weibull distribution is a reliable model which can be used to accumulate the characteristic breakdown strength of dielectrics. It is described by the following equation: ${\ln(-\ln(1-P(E)))\;=\;\beta \ln(E)-\beta \ln(E}_{b})$, where P(*E*) is the cumulative breakdown probability, *E* and *β* is the breakdown strength obtained in each test, and the Weibull modulus representing the degree of dispersion of the breakdown strength values, respectively. *E*_b_ is the breakdown strength when the cumulative breakdown probability is 63.2% [5,51,52]. As can be seen from Figure 2b, the characteristic breakdown strengths of PVTC, PMMA, 1-9, 3-7, 5-5, 7-3, and 9-1 sandwich-structure dielectrics are 195.5, 434.0, 399.1, 309.8, 297.8, 282.1, and 269.6 kV/mm, respectively. The breakdown field strength of the composite dielectric increases as the PMMA content increases. However, based on Figure 2a, it is known that an increase in the volume content of PMMA brings a decrease in permittivity, which is not beneficial for increasing energy storage density. Therefore, it is necessary to find an optimal ratio at which the composite dielectric has the highest energy storage performance.

**Figure 3:**
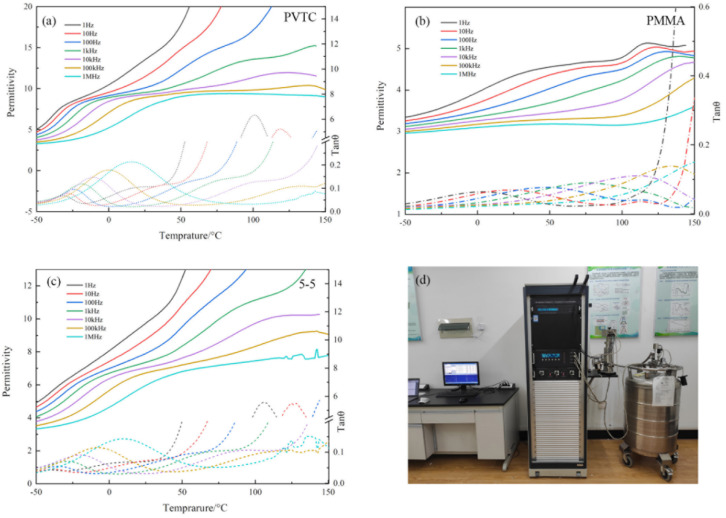
The temperature dependence of permittivity and dielectric loss for (**a**) PVTC, (**b**) PMMA, and (**c**) 5-5 Sandwich structure composite dielectric. (**d**) Digital photo of dielectric testing instrument.

To further explore the molecular chain motion of the composite dielectric, the dielectric-temperature characteristics were tested and the results are presented in Figure 3. It was reported that the glass transition of PVTC was about −40 °C [40], and a relaxation process can be clearly observed in Figure 3a, where the dielectric constant of PVTC increases rapidly due to molecular chain segment movement during the glass transition process around −40 °C accompanying a dielectric loss peak and the peak-temperature increases with the increase of measured frequency. As shown in Figure 3b, the relaxation due to the rotation of the partial rotation of −COOCH_3_ side groups of PMMA and glass transition processes were observed at 40 and 120 °C, respectively [41]. In Figure 3c, three relaxation peaks to PVTC and one relaxation peak of PMMA can be observed. Because there is a sharp permittivity and constant loss increase in the high temperature zone for PVTC due to space charge [42], the relaxation peak of PMMA indicates the glass transition process of PMMA in Figure 3c cannot be observed. Generally speaking, the relaxation peak in the sandwich-structure dielectric is a superposition of PVTC and PMMA, which indicates that the molecular chain motions between the two polymers do not affect each other.

Under the AC electric field, the distribution of electric field is inversely proportional to the permittivity of the dielectric [13]. Therefore, the ratio of the local electric field of PVTC and PMMA is 4:10, and the ratio of breakdown electric field is 195.5:434, which means that the local electric field in PMMA exceeds the breakdown electric field in advance of PVTC. That is to say, the breakdown occurs first in the PMMA layer. When the local electric field of PMMA reaches 434kV/mm, the electric field in PVTC is 173.6 kV/mm. According to their thickness ratio, the calculated breakdown field strengths of 1-9, 3-7, 5-5, 7-3, and 9-1 composite dielectric are 407.96, 355.88, 303.8, 251.72 and 199.64 kV/mm, respectively. The measured value is lower than the calculated value when the volume fraction of PMMA is greater than 50% for the following reasons: At the beginning of breakdown, a particle (electron or ion) in PMMA changes from a fundamental state to an excited state, due to the strong coupling between solid molecules which triggers the vibration of the surrounding particles resulting in the breakdown of the entire dielectric [30]. If the probability of breakdown per unit volume in PMMA is equal, the thicker the PMMA layer, the greater the probability of breakdown. From this point of view, the thicker PMMA layer leads to a slight decrease in breakdown strength.

**Figure 4:**
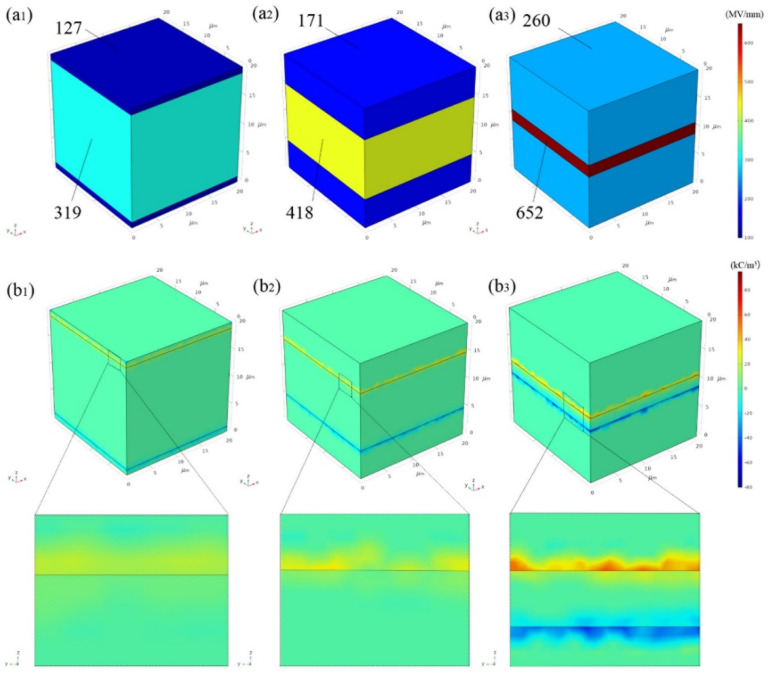
(**a_1_**–**a_3_**) Electric field distributions and (**b_1_**–**b_2_**) space charge density obtained through finite element simulation for 1-9, 5-5, and 9-1 sandwich-structure composite dielectric.

To further explore and observe the electric field and space charge distribution within the dielectric, finite element analysis simulations were performed using COMSOL Multiphysics 5.4a software. A mean electric field with a modulus of 300 kV/mm was applied from top to bottom. Here, three representative components of the composite dielectrics were simulated, namely 1-9, 3-7, and 9-1 composite dielectrics. The simulation was performed in a 100 Hz AC steady-state field. The relative dielectric constants of PVTC and PMMA are obtained from Figure 4a. The electric field borne by each layer is plotted in Figure 4. Comparing the electric field distributions of the sandwich-structure composite dielectrics with different compositions, it can be seen that the PMMA layer in the 9-1 composite dielectric withstands an electric field of 650 MW/m, which exceeds the breakdown strength of pristine PMMA. In the 1-9 dielectric, the electric field withstood by the PVTC layer and the PMMA layer are smaller. It can be seen from Figure 4b that space charge mainly exists on the PVTC side of the interface. In addition, the greater the PVTC volume fraction, the greater the space charge density, which also explains the enhancement of permittivity in 9-1. The thinner the PMMA layer, the closer the positive and negative space charges are, and the greater the electric field distortion caused, which increases the probability of a breakdown failure [47].

**Figure 5:**
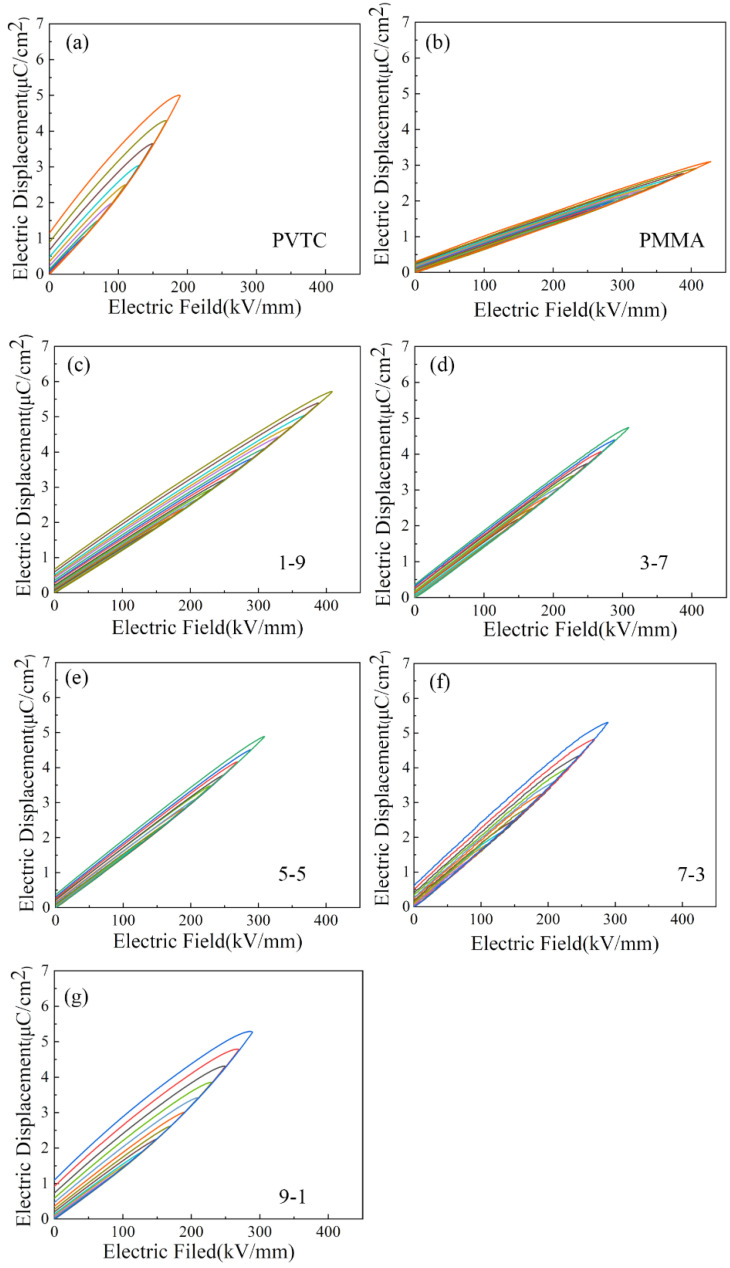
The D-E loops for (**a**) pristine PVTC, (**b**) pristine PMMA, (**c**) 1-9, (**d**) 3-7, (**e**) 5-5, (**f**) 7-3, and (**g**) 9-1 sandwich-structure PVTC/PMMA dielectric.

The D-E loops for dielectrics are tested and shown in Figure 5. It can be seen from Figure 5 that under the same electric field, the electrical displacement of pristine PVTC is largest and the electrical displacement of pristine PMMA is lowest, which is consistent with previous research. In general, the D-E loops’ shape characteristics such as maximum polarization, residual polarization, and so on, of sandwich-structure dielectrics are basically between pristine PVTC and PMMA’s. In addition, under the same electric field, the higher the volume fraction of PVTC, the greater the maximum electrical displacement; the higher the volume fraction of PMMA, the lower the residual polarization. It is worth noting that the residual polarization of 9-1 composite dielectric is even higher than that of pristine PVTC, which reflects that the loss is increased due to the relaxation polarization of the interface. For 1-9 composite dielectric, the residual polarization is very small, which is related to the small volume fraction of PVTC, but it may also be related to the thin PVTC layer and the difficulty in forming a large crystalline region [32]. In conclusion, it is effective to reduce the residual polarization of PVTC and increase the breakdown strength by constructing sandwich structure in energy storage dielectric.

**Figure 6:**
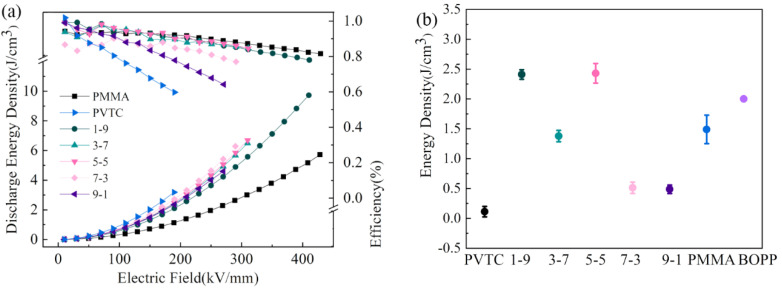
(**a**) The energy storage characteristics of composite dielectrics. (**b**) The energy density of dielectric with an energy storage efficiency of 90%.

According to D-E loops in Figure 5, the energy storage density (discharge energy density) and energy storage efficiency of the dielectric under different electric fields can be calculated and are displayed in Figure 6. The breakdown strength and polarization intensity of sandwich-structure composite dielectric are between PVTC and PMMA, and its energy storage density is greater than that of pristine dielectric. The energy storage density at maximum applied electric field of pristine PVTC, pristine PMMA, 1-9, 3-7, 5-5, 7-3, and 9-1 sandwich-structure dielectric are 3.18, 5.7, 9.7, 6.5, 6.7, 6.3, and 4.6 J/cm^3^, respectively. The charge-discharge efficiency is 60%, 81.5%, 77.8%, 84.7%, 84.5%, 77.0%, and 64.2%, respectively. The efficiency of energy-storage dielectrics are important for their applications. The energy-storage efficiency of commercial BOPP dielectric is higher than 90% [4]. Therefore, this work focuses on the energy storage density of sandwich-structure composite dielectric when the energy storage efficiency is 90%, which is shown in Figure 6b. As can be seen from Figure 6b, when the energy storage efficiency of the 1-9 sandwich-structure composite dielectric is 90%, the energy storage density reaches 2.569 J/cm^3^, which is higher than BOPP (2 J/cm^3^) [53,54,55]. The energy storage density under high energy storage efficiency (90%) of similar work is summarized and compared in Table 2 [13,18,37,49,56,57,58,59].

## 4. Conclusions

In this work, a series of sandwich-structure PVTC/PMMA composite dielectrics with different volume fractions have been successfully prepared by electrostatic spinning. The dielectric and breakdown performance shows that the sandwich structure can combine the advantages of different dielectrics and improve the comprehensive performance of energy-storage dielectric. Among them, the 1-9 sandwich-structure PVTC/PMMA composite dielectric has an excellent energy density, reaching 9.7 J/cm^3^ under an electric field of 400 kV/mm. Compared with the current commercial capacitor dielectric BOPP, the energy storage density is increased by 25%. This study provides the experience and theory supporting hierarchical structure and a new design method of high-performance energy storage dielectrics, which is conducive to the development of the next generation of dielectric capacitors.

## Figures and Tables

**Table 1:** The volume of the precursor used in electrostatic spinning ^1^.

V_PVTC_:V_PMMA_	V_PVTC_ for First Layer /mL	V_PMMA_ for Second Layer /mL	V_PVTC_ for Third Layer /mL
1:9	0.3125	1.125	0.3125
3:7	0.9360	0.875	0.9360
5:5	1.5625	0.625	1.5625
7:3	2.1875	0.375	2.1875
9:1	2.8125	0.125	2.8125

^1^ ρ(PMMA): 1.88 g/mL, ρ(PVTC): 1.78 g/mL.

**Table 2:** Comparison with published work.

Sample	Discharge Energy Density(J/cm^3^) @ 90% Efficiency	Reference
1 vol % PDA-SiO_2_@BT NPs/PVDF	2.1	[13]
PVDF/PS-b-PMMA composite	1	[18]
BCZT + Ag@Al_2_O_3_/PMMA/PVDF	0.8	[37]
5 vol % BZT-BCT NFs/PVDF composite	0.9	[49]
P(VDF-TrFE-CFE)/PI bilayer films	1	[56]
15 vol % 4L BT/P(VDF-HFP) composites	1	[57]
BOPVDF films	2	[58]
25 vol % sandwich-structured PVDF/P(VDF-TrFE-CTFE)/PVDF	1	[59]
1-9 Sandwich Structure PVTC/PMMA composite	2.569	This work
